# Effects of Time Constraints and Goal Setting on Basketball Shooting

**DOI:** 10.3389/fpsyg.2022.923061

**Published:** 2022-06-29

**Authors:** Jason Kostrna

**Affiliations:** Department of Teaching and Learning, Florida International University, Miami, FL, United States

**Keywords:** attention, anxiety, kinematics, motor control, pressure

## Abstract

In sport, numerous high-pressure situations require athletes to perform motor tasks under temporally constrained circumstances. The present study investigated the effects of time constraints on anxiety, attention, performance, and mechanics of basketball free-throw shooting. Additionally, the potential benefits of goal setting were examined in relation to performance in time-constrained situations. Forty undergraduates (*n* = 10 elite basketball players, *n* = 15 experienced, *n* = 15 inexperienced) attempted free throws in timed, untimed, and goal-oriented conditions. In the timed condition, participants attempted to make as many field goals as possible in 30 s. In the untimed condition, participants attempted the same number of field goals as they did in the timed trial but without a time constraint. In the goal-oriented condition, participants attempted to surpass their highest number of successful field goals while once again under a 30-s time constraint. Participants in the timed condition had the worst field goal percentage (*M* = 45.20%, *SD* = 21.96%), while the untimed (*M* = 55.76%, *SD* = 21.12%, *p* < 0.05, *d* = 0.49) and goal-oriented conditions (*M* = 55.79%, *SD* = 22.92%, *p* < 0.05, *d* = 0.47) had similar field goal percentages. In addition, joint consistency in the elbow and knee increased during the untimed condition compared to both timed and goal-oriented conditions. Results indicate that a goal-oriented focus may prevent performance declines present in time-constrained situations.

## Introduction

In sport competitions, games, seasons, careers, and millions of dollars can come down to the performance of individual athletes in a few brief moments (e.g., Gröpel and Mesagno, 2019). These moments are high-stakes, temporally constrained, and often perceived as highly stressful (Janelle and Hillman, 2003). In these situations, emotions and performance can change rapidly (e.g., Gröpel and Mesagno, 2019; Lee et al., 2019). To succeed, athletes must handle the emotional and temporal demands of these high-pressure moments (e.g., Gröpel and Mesagno, 2019).

Researchers examining emotional and attentional responses in late game moments have predominantly focused on either emotional regulation or psychological skills (Janelle and Hillman, 2003). Emotional regulation is essential to achieve peak performance in high-pressure moments and has received extensive research attention (Laborde et al., 2015; Kopp and Jekauc, 2018). To understand emotional regulation in high-pressure moments, researchers have examined choking, a sub-optimal performance in highly stressful environments, for several decades (cf., Gröpel and Mesagno, 2019).

Researchers studying choking have predominantly examined two main theories: explicit monitoring and distraction (Englert and Oudejans, 2014; Masaki et al., 2017; Gröpel and Mesagno, 2019). Distraction theory suggests that an athlete’s working memory is finite, and that in stressful situations it is divided between the task-irrelevant distractor and the task-relevant performance (Beilock et al., 2004). Thus, in high-pressure situations, distractions increase dissociative attention (often toward thoughts associated with anxiety and worry) leading to decreased cognitive resources available for task execution, eventually resulting in suboptimal performance (Englert and Oudejans, 2014; Masaki et al., 2017). Alternatively, explicit monitoring theory suggests that pressure situations raise self-consciousness awareness, resulting in increased associative attention to somatic perceptions, and motor processes which would otherwise be automatic (e.g., Beilock and Carr, 2001; Beilock et al., 2004; Lee et al., 2019). In support of explicit monitoring theory, the majority of sport performance research indicates that directing attention toward a single, task-relevant, dissociative cue is advantageous for performance (e.g., Beilock et al., 2004; Law and Wong, 2021). Moreover, research on sport performance and attention indicates that a task-relevant dissociative focus improves biomechanical properties as well (e.g., Zachry et al., 2005; Lohse et al., 2010; Schücker and Parrington, 2019).

These changes in attention affect motor skill execution *via* the phenomenon of dechunking and freezing degrees of freedom (e.g., Higuchi et al., 2002; Verstynen et al., 2012). Dechunking refers to when an athlete focuses on individual pieces, or chunks, of their mechanics learned during nascent stages of their skill acquisition, preventing a natural and automatic execution of the task (e.g., Bellomo et al., 2018). Studies on dechunking typically instruct participants to focus their attention on movement chunks, similar to those found during high anxiety and earlier stages of learning, and report that movement variability increases with associative attention (Gray, 2004; Toner and Moran, 2011; Land and Tenenbaum, 2012; Verstynen et al., 2012; Bellomo et al., 2018). Furthermore, this increase in variability has led to performance declines in motor tasks (Gray, 2004; Toner and Moran, 2011; Land and Tenenbaum, 2012). Freezing degrees of freedom resulting from increased muscle tension limits mobility in joints and disrupts task performance (e.g., Higuchi et al., 2002). This pattern of freezing degrees of freedom explains the increased muscle tension and performance declines found in studies of associative attention and stress during athletic performance (e.g., Neumann and Heng, 2011; Law and Wong, 2021). These two theories of movement patterns during high anxiety, dechunking (resulting in greater movement variability), and freezing degrees of freedom (resulting in greater movement variability) offer two contradictory predictions of how anxiety influences motor behavior.

The addition of time constraints to the these high-pressure situations place further demands on cognitive and motor processes (e.g., Gröpel and Mesagno, 2019). In particular, time demands on motor tasks result in the well-studied, speed-accuracy tradeoff (e.g., Fitts, 1954; Muhs et al., 2018). When the ability to successfully complete a task is challenged by time constraints, individuals are forced to make decisions which balance the speed and accuracy of their motor execution (Freeston and Rooney, 2014; Onagawa et al., 2019). This decision-making draws upon finite attentional, cognitive, and motor resources that are needed for task execution (Spieser et al., 2016; Pooripanyakun et al., 2020). Moreover, under temporal constraints, these limited resources may already be devoted to debilitative emotional states like anxiety (e.g., Beilock et al., 2004). The continual division of attentional, cognitive, and motor processes, resulting from temporal constraints and debilitative emotional states, often results in suboptimal performance (Englert and Oudejans, 2014).

Interventions to improve performance and reduce anxiety in high-stress situations implement a range of psychological skills (e.g., visualization, self-talk, cognitive restructuring; Gröpel and Mesagno, 2019). Among these interventions, goal setting has received support through the application of achievement goal theory (cf., Senko et al., 2011; Jeong et al., 2021). Interventions using approach forms of goal setting have found reductions in competitive anxiety and improved performance for high-level soccer players, field hockey players, boxers, and golfers (Kingston and Hardy, 1997; Jackson et al., 2006; O’Brien et al., 2009). In contrast, avoidance goals are correlated with increased anxiety and performance declines (Senko et al., 2011). The effects of goal-setting-interventions for less-skilled participants have been less consistent. Goal-setting improved performance and anxiety levels in novice putters (e.g., Goudas et al., 1999; Jeong et al., 2021), but produced null results the performance of non-elite boxers and drivers (O’Brien et al., 2009; Mullen et al., 2015; Jeong et al., 2021). Therefore, this study examines skill-level as a potential moderator for the effects of approach forms of goal setting on anxiety and sport performance.

The main purposes of this study were to examine the effects of time pressure on performance and to test if goal setting can improve performance under time pressure. A primary hypothesis of the study was that under time pressure, implementing an approach form of goal setting would increase basketball field goal percentage, especially for experienced and elite participants. Relatedly, it was hypothesized that, novice participants would experience the highest levels of anxiety, particularly, during the timed and goal-oriented conditions. Novice participants should have the least consistent shooting kinematics, and time pressure should increase variations in shooting kinematics of major muscle groups. Finally, an approach form of goal setting should promote more consistent kinematics despite the time pressure.

## Materials and Methods

### Participants

Researchers recruited 40 participants (all male) from two southeastern United States universities. Participants were recruited *via* flyer and word of mouth from campus recreation facilities and university basketball teams. Ten participants were members of men’s National Collegiate Athletic Association (NCAA) division 1 basketball teams, comprising an elite skill group. Fifteen participants reported playing at least 2 years of officiated or competitive basketball (see Table 1), forming an experienced group. All participants in this skill group reported playing some level of competitive basketball within the last 2 years. Fifteen participants reported playing less than 2 years of competitive basketball, forming the novice group. The present study used only male participants due to a limited participant pool, differing shooting kinematics between genders, and logistical difficulties related to differing basketball sizes.

**TABLE 1:** Demographic information by skill level.

Skill level	Age	Years of competitive basketball experience	Height (m)
Novice	22.00 (2.59)	0.13 (0.35)	1.81 (.07)
Experienced	22.27 (3.49)	8.00 (4.19)	1.84 (.08)
Elite	20.30 (0.82)	13.00 (3.02)	1.92 (.07)

### Design

The present study conformed to a two-way, repeated-measures design. Skill level was used as a between-subjects factor, and time condition as a within-subject’s factor. Each participant attempted from the free-throw line in three time conditions; timed, untimed, and goal-oriented. In the timed condition, participants were tasked with successfully scoring as many field goals as possible in 30 s (standard NCAA shot clock). For the untimed condition, participants attempted the same number of field goals that they took in the timed trials but without the 30-s time constraint. Finally, for the goal-oriented condition, researchers reminded each participant of the number of successful field goals from the untimed condition and informed them that they needed to surpass that number of successful field goals while under the 30-s constraint. The order of trials was kept consistent in order to meet the requirements of the three conditions. Although free-throw shooting in a game situation is not under time pressure, its standardized range and difficulty make it a repeatable phenomenon to study and extrapolate to all basketball shooting, which is frequently under time pressure.

### Measures

The dependent variables were field goal percentage (i.e., number of field goals scored divided by the number of field goals attempted, times 100), anxiety, attention, maximum joint angles, and variability of maximum joint angles.

#### Anxiety

The Anxiety Thermometer was used to assess participants’ perceived state of anxiety. The Anxiety Thermometer uses a 10-point Likert-type scale where 1 is *not at all anxious/nervous* and 10 is *extremely anxious/nervous*. The Anxiety Thermometer has been successfully used in sport contexts and provides repeatable and validated results (correlation coefficients between 0.60 and 0.78; e.g., Nieuwenhuys and Oudejans, 2011).

#### Attention

Attention was measured periodically using a 10-point scale ranging from 0 (*external thoughts, daydreaming, environment, singing songs)* to 10 (*internal thoughts, how body feels, breathing, muscles)*. The Tammen (1996) attention scale was originally designed to represent the continuum of attention strategies using a scale from 0 (*pure dissociation*) to 10 (*pure association)*. A one-question scale has been repeatedly used by researchers to quickly collect association or dissociation attention data during physical activity (e.g., Masters and Ogles, 1998; Hutchinson et al., 2018).

#### Kinematic Analysis

To analyze the two-dimensional sagittal plane videos, researchers using Dartfish 6 (Swiss Federal Institute of Technology, Lausanne, Switzerland) software, went frame by frame through each field goal attempt and recorded joint angles for maximum knee flexion and maximum elbow flexion. Furthermore, forearm angle, relative to the ground, at ball release was recorded (Mullineaux and Uhl, 2017; Ogawa et al., 2019). Finally, researchers measured shooting duration as the time between maximum knee flexion and ball release. This definition of shooting duration was selected as it represented the upward motion of the shot and was independent of any pauses in pre-shot routine. Joint angles and shooting durations from each attempted field goal within a time condition were then aggregated to provide averages and standard deviations of each joint and shooting duration for each of the three time conditions (Mullineaux and Uhl, 2017).

### Apparatus

Each participant attempted field goals from an NCAA men’s free-throw line at a regulation basket in the university’s arena using men’s official size basketballs. Twenty balls were conveniently located in a ball cart on the participants’ non-dominant side. A laptop, running MediaLab v. 2010, was used to record participants’ demographics and self-reported skill level as well as anxiety level and attentional focus after each trial. The laptop, easily visible to participants located under the basket, displayed the 30-s shot clock that was used for the timed conditions. Researchers placed a Sony Handycam HDR-XR160 (60 frames per second) on the free-throw line to record a sagittal view of the entire field goal attempt. A second identical camera was placed beyond the three-point line and just off the participants’ dominant shoulder to record the ball trajectory and result.

### Procedure

After signing the informed consent and answering several questions about basic demographic information regarding their basketball experience, participants took as many practice field goals as they felt they needed. After participants completed their warmup, researchers explained the Anxiety Thermometer and attention scale before informing participants that they had 30 s to make as many free throws as they could. Researchers then told participants to begin shooting and started the clock on the laptop (i.e., timed trial). After completing the timed trial, participants reported their anxiety level and attention on the laptop, while researchers refilled the ball cart. Next, researchers told participants the number of field goals they attempted and instructed them to attempt the same number of field goals again but without the 30-s shot clock (i.e., untimed trial). Again, participants reported their anxiety level and attention after completing the trial. Researchers then informed participants that the number of field goals scored in the timed or untimed condition (which ever was higher) would represent the goal for their next timed condition. Participants were given the goal to make more field goals than they had in either condition while under the 30-s time constraint. After the goal-oriented trial, participants reported their anxiety level and attention. Researchers then debriefed participants on the purpose of the study.

### Statistical Analysis

To examine the effects of time pressure and goal setting on field goal percentage, anxiety, attention, and kinematic variables, a series of repeated measures MANOVAs were conducted. The first MANOVA analyzed field goal percentage, anxiety, and attention, while the second analyzed kinematic variables. Initial screening of the data for assumptions required for repeated measures MANOVAs found skewed distributions (skewness/standard error and kurtosis/standard error greater than 1.96) for several dependent variables (particularly, average joint angles). However, the data did not violate any other assumptions for repeated-measures MANOVA or subsequent univariate analyses (e.g., no outliers, Mauchly’s test of sphericity *p* > 0.05). As such, the possibility of log transforming the data was considered to normalize the distribution. However, with the knowledge that the other assumptions had been met, the main benefit of such a transformation was a slight increase in statistical power at the cost of ease of interpretation (cf., Pek et al., 2018). Moreover, after a review of log-transformed data, the loss of power resulting from analyzing the untransformed data did not impact any of the significant findings. Therefore, the data were analyzed using original values despite several dependent variables having non-normal distributions. Effect sizes were calculated using η*_*p*_*^2^ and Cohen’s *d* (using pooled standard), and Bonferroni adjustments were applied on *post hoc* analyses.

## Results

### Field Goal Percentage and Psychological Analysis

A two-way repeated-measures MANOVA on field goal percentage and psychological variables indicated a non-significant interaction of time condition and skill level, Wilks’ λ = 0.56, *F*_(12, 64)_ = 1.77, *p* = 0.07, η*_*p*_*^2^ = 0.25. Given the near significance of this result and the importance of these dependent variables, further analyses of this interaction were conducted; however, any interpretations should be tempered by potential Type I errors. Significant main effects of time condition, Wilks’ λ = 0.27, *F*_(6, 32)_ = 14.34, *p* < 0.05, η*_*p*_*^2^ = 0.73 and skill level, Wilks’ λ = 0.54, *F*_(6, 70)_ = 4.26, *p* < 0.05, η*_*p*_*^2^ = 0.27, were found.

#### Field Goal Percentage

**FIGURE 1:**
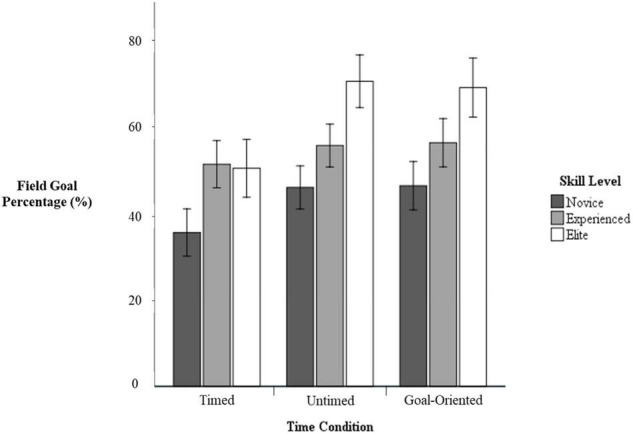
Shooting percentage by skill level and time condition with 95% confidence intervals.

Univariate analysis found no significant interaction between skill level and time condition, *F*_(4, 74)_ = 0.64, *p = 0*.64, η*_*p*_*^2^ = 0.03. However, there was significant main effect of time condition, *F*_(2, 74)_ = 4.97, *p* < 0.05, *η_*p*_^2^* = 0.12. Specifically, participants had lower field goal percentages in the timed condition (*M* = 45.20%, *SD* = 21.96%) than in the untimed (*M* = 55.76%, *SD* = 21.12%, *p* < 0.05, *d* = 0.49) and goal-oriented conditions (*M* = 55.79%, *SD* = 22.92%, *p* < 0.05, *d* = 0.47). There was no significant difference between untimed and goal-oriented conditions (*p* = 0.99, *d* = 0.00). There was also a significant main effect of skill level on field goal percentage, *F*_(2, 37)_ = 6.70, *p* < 0.05, η*_*p*_*^2^ = 0.27. Elite participants had significantly higher field goal percentages (*M* = 63.36%, *SD* = 23.06%) than novice participants (*M* = 42.64%, *SD* = 20.70%, *p* < 0.05, *d* = 0.95), but not experienced participants (*M* = 54.46%, *SD* = 19.85%, *p* = 0.39, *d* = 0.41). There was no significant difference between novice and experienced participants (*p* = 0.08, *d* = 0.58; see Figure 1).

Importantly, for the interpretation of field goal percentage, a separate MANOVA with field goal attempts in the timed and goal-oriented condition as dependent variables found no interaction between skill level and condition, *F*_(2, 37)_ = 0.79, *p* = 0.46, η*_*p*_*^2^ = 0.04. There was a significant main effect of condition, *F*_(1, 37)_ = 7.75, *p* < 0.05, η*_*p*_*^2^ = 0.17, such that participants attempted fewer field goals in the timed condition (*M* = 8.65, *SD* = 2.32) than the goal-oriented conditions (*M* = 9.28, *SD* = 2.46, *d* = 0.26). Additionally, there was a significant effect of skill level, *F*_(2, 37)_ = 4.21, *p* < 0.05, η*_*p*_*^2^ = 0.19, indicating that elite participants attempted fewer field goals (*M* = 7.30, *SD* = 2.62) than experienced participants (*M* = 9.63, *SD* = 2.19, *p* < 0.05, *d* = 0.96), but not novice participants (*M* = 9.40, *SD* = 1.58, *p* = 0.06, *d* = 0.97). There was no significant difference between novice and experienced participants (*p* > 0.99, *d* = 0.12).

#### Anxiety

**FIGURE 2:**
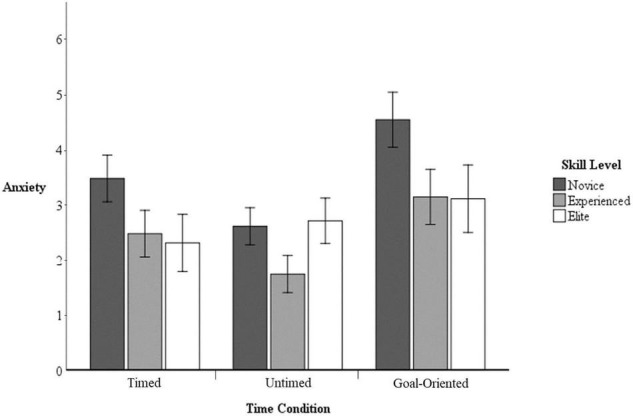
Anxiety by skill level and time condition with 95% confidence intervals.

Univariate analysis revealed no significant interaction between skill level and time condition on anxiety levels, *F*_(4, 74)_ = 1.33, *p = 0.*27, η*_*p*_*^2^ = 0.07. However, a significant main effect of time condition was found, *F*_(2, 74)_ = 9.45, *p* < 0.05, η*_*p*_*^2^ = 0.20. Participants reported higher anxiety in the goal-oriented condition (*M* = 3.65, *SD* = 2.01) than in the timed (*M* = 2.80, *SD* = 1.68, *p* < 0.05, *d* = 0.46) and untimed conditions (*M* = 2.30, *SD* = 1.34, *p* < 0.05, *d* = 0.79). There was no significant difference in the timed and untimed conditions (*p* = 0.51, *d* = 0.32). The main effect of skill level on anxiety was just barely not significant, *F*_(2, 37)_ = 3.18, *p* = 0.06, η*_*p*_*^2^ = 0.14. Descriptively, novice participants reported the most anxiety (*M* = 3.53, *SD* = 1.82) followed by elite participants (*M* = 2.70, *SD* = 1.43) and experienced participants (*M* = 2.44, *SD* = 1.75; see Figure 2).

#### Attention

**FIGURE 3:**
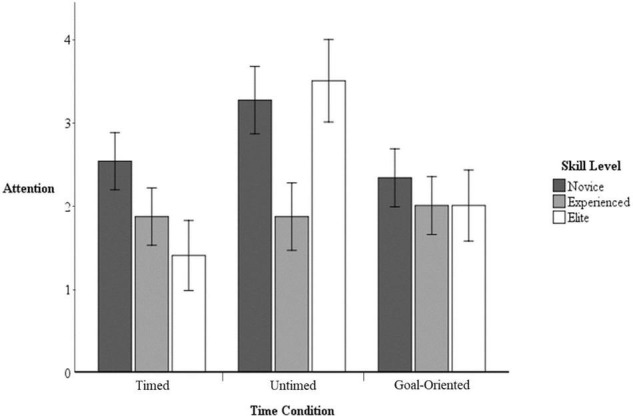
Attention by skill level and time condition with 95% confidence intervals.

Univariate analysis indicated a significant interaction between skill level and time condition on attention, *F*_(4, 74)_ = 2.99, *p* < 0.05, η*_*p*_*^2^ = 0.14. Driving this interaction is the experienced participants’ more dissociative attention during the timed condition than other groups in comparison to the timed or goal-oriented conditions (see Figure 3). Overall, participants in the untimed condition reported more associative attention than in the other two conditions; however, the experienced group maintained more dissociative attention than any other group. Further contributing to this interaction is the experienced participants’ consistent attentional focus across all three time conditions.

### Kinematic Analysis

A two-way repeated-measures MANOVA of kinematic variables (i.e., joint angles, joint variance, and movement time) did not indicate a significant interaction between time condition and skill level, Wilks’ λ = 0.34, *F*_(32, 44)_ = 0.98, *p* = 0.52, η*_*p*_*^2^ = 0.42. However, there was a significant main effect of time condition, Wilks’ λ = 0.20, *F*_(16, 22)_ = 5.37, *p* < 0.05, η*_*p*_*^2^ = 0.80 (see Table 2), as well as of skill level, Wilks’ λ = 0.33, *F*_(16, 60)_ = 2.77, *p* < 0.05, η*_*p*_*^2^ = 0.42 (see Table 3).

**TABLE 2:** Means and standard deviations of average maximum joint angle and standard deviation of maximum joint angle by joint and time condition.

Joint	Condition	Average angle (°)	Standard deviation of angles (°)
Maximum knee flexion	Timed	115.99 (13.06)^a^	4.03 (2.05)^a^
	Untimed	112.25 (12.19)^b^	3.06 (1.33)^b^
	Goal-oriented	117.20 (12.58)^a^	4.04 (1.45)^a^
Maximum elbow flexion	Timed	65.14 (10.49)^a^	3.19 (1.87)^a^
	Untimed	65.06 (10.80)^a^	2.57 (1.02)^a^
	Goal-oriented	65.81 (10.69)^a^	3.06 (1.35)^b^
Forearm angle at release	Timed	69.04 (13.58)^a^	3.52 (1.54)^a^
	Untimed	69.57 (13.29)^a^	3.02 (1.71)^a^
	Goal-oriented	67.70 (13.17)^b^	3.15 (1.53)^a^

*^ab^Within a column and a joint, means without a common superscript defer, p < 0.05.*

**TABLE 3:** Means and standard deviations of average maximum joint angle and standard deviation of maximum joint angle by joint and skill level.

Joint	Skill level	Average angle (°)	Standard deviation of angles (°)
Maximum knee flexion	Novice	112.46 (14.56)^a^	4.17 (1.71)^a^
	Experienced	116.61 (12.74)^a^	4.00 (1.66)^a^
	Elite	116.99 (8.66)^a^	2.58 (1.18)^b^
Maximum elbow flexion	Novice	69.10 (10.63)^a^	3.31 (1.55)^a^
	Experienced	62.44 (12.30)^a^	2.88 (1.36)^a^
	Elite	64.03 (4.60)^a^	2.47 (1.38)^a^
Forearm angle at release	Novice	60.40 (10.68)^a^	3.88 (1.45)^a^
	Experienced	70.50 (13.36)^a^	3.18 (1.78)^a^
	Elite	78.73 (7.83)^b^	2.33 (1.00)^b^

*^ab^Within a column and a joint, means without a common superscript defer, p < 0.05.*

#### Joint Angles

##### Average Angles

For maximum knee flexion, univariate analysis revealed a significant effect of time condition, *F*_(2, 74)_ = 12.02, *p* < 0.05, η*_*p*_*^2^ = 0.25. Kinematics in the untimed condition indicated significantly more flexing of the knee than in the timed condition (*p* < 0.05, *d* = 0.30) or goal-oriented condition (*p* < 0.05, *d* = 0.41). There was no significant difference between knee angles in the timed and goal-oriented conditions (*p* = 0.53, *d* = 0.10). There was no significant effect of skill level on maximum knee flexion *F*_(2, 37)_ = 0.59, *p* = 0.56, η*_*p*_*^2^ = 0.03.

Maximum elbow flexion did not significantly differ across time condition, *F*_(2, 74)_ = 1.66, *p* = 0.20, η*_*p*_*^2^ = 0.04, or skill level, *F*_(2, 37)_ = 1.65, *p* = 0.21, η*_*p*_*^2^ = 0.08.

Univariate analysis of forearm-release angle revealed a significant effect of time, *F*_(2, 74)_ = 9.18, *p* < 0.05, η*_*p*_*^2^ = 0.20, such that the goal-oriented condition resulted in a lower forearm angle at release than the timed (*p* < 0.05, *d* = 0.10) and untimed conditions (*p* < 0.05, *d* = 0.13). No significant difference was found between timed and untimed condition forearm-release angles (*p* = 0.82, *d* = 0.03). There was a significant effect of skill, *F*_(2, 37)_ = 8.123, *p* < 0.05, η*_*p*_*^2^ = 0.31 with novices having significantly lower forearm angles at release than elite participants (*p* < 0.05, *d* = 1.96). However, there was no significant difference between novices and experienced participants (*p* = 0.06, *d* = 0.84), or between experienced and elite participants (*p* = 0.25, *d* = 0.75).

##### Joint Variation

Univariate analysis of standard deviation of maximum knee-flexion angle revealed a significant main effect of time condition, *F*_(2, 74)_ = 7.45, *p* < 0.05, η*_*p*_*^2^ = 0.17, such that knee angles in the untimed condition were more consistent than in both the timed condition (*p* < 0.05, *d* = 0.50) and goal-oriented condition (*p* < 0.05, *d* = 0.70). There was no significant difference between the timed condition and the goal-oriented condition (*p* = 0.99, *d* = 0.07). Additionally, knee-flexion variation significantly differed by skill level, *F*_(2, 37)_ = 6.71, *p* < 0.05, η*_*p*_*^2^ = 0.27. Elite participants had significantly less variation in their knee angle than experienced (*p* < 0.05, *d* = 0.99) or novice participants (*p* < 0.05, *d* = 1.09). There was no significant difference between novice and experienced participants (*p* = 0.99, *d* = 0.11).

A significant effect of time condition on standard deviation of maximum elbow flexion was found, *F*_(2, 74)_ = 3.66, *p* < 0.05, η*_*p*_*^2^ = 0.09, indicating that the elbow angles in the untimed condition had more consistency than those in the goal-oriented condition (*p* < 0.05, *d* = 0.45) but not those in the timed condition (*p* < 0.10, *d* = 0.33). Standard deviation of elbow-joint angle did not differ between the goal-oriented and the timed conditions (*p* < 0.99, *d* = 0.03). Analysis did not find a significant difference in standard deviation of maximum elbow flexion among skill levels, *F*_(2, 37)_ = 1.64, *p* = 0.21, η*_*p*_*^2^ = 0.08.

Univariate analysis of standard deviation of forearm angle at release did not reveal a significant difference for time condition, *F*_(2, 74)_ = 2.70, *p* = 0.07, η*_*p*_*^2^ = 0.08, but did reveal a significant main effect for the variation of forearm angle at release for skill level, *F*_(2, 37)_ = 4.11, *p* < 0.05, η*_*p*_*^2^ = 0.18. Specifically, elite participants had significantly more consistent release angles than novice participants (*p* < 0.05, *d* = 1.07) but not more than experienced participants (*p* < 0.38, *d* = 0.65). There was no significant difference between novice and experienced participants in standard deviation of forearm-release angle (*p* = 0.47, *d* = 0.43).

#### Shooting Duration

Univariate analysis on average shooting duration did not find a significant main effect of time condition, *F*_(2, 74)_ = 1.96, *p* = 0.45, η*_*p*_*^2^ = 0.05, but did reveal a significant main effect of skill level, *F*_(2, 37)_ = 8.97, *p* < 0.05, η*_*p*_*^2^ = 0.33. Elite participants had longer shooting durations (*M* = 0.57 s, *SD* = 0.35 s) than both novice (*M* = 0.26 s, *SD* = 0.05 s, *d* = 1.22, *p* < 0.05) and experienced participants (*M* = 0.39 s, *SD* = 0.19 s, *d* = 0.66, *p* < 0.05). There was no significant difference between novice and experienced participants (*d* = 0.86, *p* = 0.21).

Conversely, the standard deviation of shooting duration differed by time condition, *F*_(2, 74)_ = 5.85, *p* < 0.05, η*_*p*_*^2^ = 0.14, and skill level, *F*_(2, 37)_ = 3.71, *p* < 0.05, η*_*p*_*^2^ = 0.17. Shooting durations in the untimed condition (*M* = 0.06, *SD* = 0.10) were significantly less consistent than those in the goal-oriented condition (*M* = 0.03, *SD* = 0.03, *d* = 0.45, *p* < 0.05) but not less than those in the timed condition (*M* = 0.04, *SD* = 0.04, *d* = 0.34, *p* = 0.07). Furthermore, there was no significant difference between the goal-oriented and timed conditions (*d* = 0.17, *p* = 0.99). Additionally, elite participants had higher standard deviations of shooting durations (*M* = 0.07, *SD* = 0.09) than novice participants (*M* = 0.02 = *SD* = 0.02, *d* = 0.23, *p* < 0.05) but not higher than experienced participants (*M* = 0.05, *SD* = 0.07, *d* = 0.66, *p* = 0.29). There was no difference between novice and experienced participants (*d* = 0.51, *p* = 0.79).

## Discussion

The present study examined the effects of time pressure on basketball shooting. Overall, field goal percentage was lowest in the timed condition. However, as hypothesized, the goal-oriented condition improved field goal percentages to a similar level as the untimed condition. Thus, the addition of approach-oriented, goal setting offset the negative impact of time constraints on motor execution. Furthermore, although goal setting slightly elevated anxiety levels compared to the timed condition, there was no indication of suboptimal performance representative of choking (e.g., Beilock and Carr, 2001; Masaki et al., 2017). Therefore, the present investigation supported previous research that goal setting improves performance in potentially stressful situations (Gröpel and Mesagno, 2019).

The beneficial impact of goal setting on field goal percentage was particularly efficacious for elite participants whose field goal percentages improved the most from timed to goal-oriented conditions. A similar differential impact of goal setting on anxiety for novice participants was observed with novice participants experiencing the greatest increases in anxiety while in the goal-oriented condition. Thus, the present study supports previous research that skill level moderates the effects of goal setting on performance and anxiety. However, the current findings deviate from previous research by finding increased anxiety levels among all groups in the goal-oriented condition (e.g., Jeong et al., 2021).

This study’s examination of the mediating role of attention in anxiety and performance did not support either explicit monitoring and distractions hypotheses (Englert and Oudejans, 2014; Masaki et al., 2017; Gröpel and Mesagno, 2019). The time allowance in the untimed condition reduced anxiety and avoided the speed-accuracy tradeoff resulting in a higher field goal percentage than the timed condition. The timed condition introduced the speed-accuracy tradeoff, increased anxiety level, and dissociated attention culminating in lower field goal percentages. The addition of goal setting further dissociated attention and increased anxiety but returned field goal percentages to untimed levels of performance. Therefore, although goal setting improved performance, there was no clear relationship between anxiety, attention, and performance. Since the present study did not result in high levels of anxiety or induce suboptimal performance, explicit monitoring and distractions hypotheses may be insufficient for explaining more modest changes in anxiety and performance. Future research should investigate the mechanisms between anxiety, attention, and performance in temporally constrained, motor tasks.

Analysis of shooting kinematics revealed that time pressure resulted in higher anxiety and less consistent knee- and elbow-flexion angles. Importantly, participants’ shot times did not change as a result of time condition. Therefore, time pressure increased anxiety resulting in decreased movement efficiency *via* inconsistent knee and elbow flexion but did not increase shot duration, culminating in suboptimal performance. This finding supports the negative impact of anxiety on movement efficiency found in previous sport research (e.g., Neumann and Heng, 2011; Bellomo et al., 2018).

While the introduction of goal setting resulted in higher field goal percentages it did not return knee and elbow flexion to the more consistent angles present during the untimed condition. Furthermore, goal setting did not dissociate attention as predicted. Despite goal setting improving performance and the consistency of kinematics, the particular mechanism for the positive effect of goal setting remains unclear. This increase in performance without change in angle consistency is potentially attributed to practice effects. The goal-oriented condition necessarily followed the other two conditions, allowing participants additional practice before participating in the goal-oriented condition. To control for this, participants attempted numerous field goals during the warmup (typically more than 20) and did not begin trials until they were comfortable with the task. Furthermore, this potential explanation is challenged by a lack of significant difference in novice field goal percentage between timed and goal-oriented conditions. Novice shooters would be the most likely to benefit from additional practice as the shooting task is common for experienced players. Similarly, there was no significant difference between joint angles in the time- and goal-oriented conditions for participants of all skill levels.

Comparing skill levels, elite participants had more consistent joint angles (i.e., knee- and forearm-release angle) and higher field goal percentages. This supports some of the previous research on performance and joint variability indicating that more consistent, gross-motor movements result in superior performance (e.g., Toner and Moran, 2011). However, other research contradicts this finding, reporting that high-skill level performers had increased movement variation allowing for increased adaptability and a higher level of performance (e.g., Neumann and Heng, 2011; Law and Wong, 2021). A possible explanation for these contradictory findings is that the particular movements analyzed may moderate the effect of skill level on joint variation. High-skill level performers may benefit from consistency of certain joints and movements (e.g., knee and release angle) while other joints are freed to move in more adaptable and reactive ways (e.g., wrist and finger articulation). Future research should examine this potential moderation.

In untimed trials, joint consistency was highest while attention was the most associative. Thus, untimed trials allowed participants time to associate attention to their shooting, resulting in more consistent movement patterns, despite a potential decrease in efficiency as a result of explicit monitoring (e.g., Lohse et al., 2010; Schücker and Parrington, 2019). However, this area of research requires further investigation as other studies have found that associative attention may result in less consistent motor movements (e.g., Lee et al., 2019). Therefore, the effect of attention on movement variability may be similarly moderated by joint location such that, under pressure, certain joints may become more variable while others become more frozen and less variable.

The present study is the first to explore the effects of time pressure and goal setting in time-constrained basketball shooting. However, several methodological limitations exist in the present study; for example, external validity is limited by an entirely male, college aged, and voluntary sample from two southeastern universities. Regarding internal validity, it is difficult to determine the specific causal mechanisms for the effects of time constraints on field goal percentage. As athletes attempt to cope with time related demands, they are required to make decisions about speed and accuracy. This makes separating the effects of anxiety and attentional changes from the speed-accuracy tradeoff difficult. It appears that different skill levels used different strategies to successfully score the highest number of field goals with elite athletes attempting fewer field goals (with longer shooting durations) than both novice and experienced participants. It is unclear if this strategic difference is due to anxiety or other elements of the speed-accuracy tradeoff. However, it is worth noting that when given a goal-oriented focus all three groups increased the number of field goals attempted indicating a similar strategy in the most situationally demanding condition. Thus, the present study does not fully separate the confounding explanations of speed-accuracy tradeoff and time-pressure’s impact on anxiety, attention, and performance. Regarding goal setting, the present study did not measure goal acceptance, therefore the level of engagement participants had with the goal setting intervention is unknown. Anecdotally participants appeared to be highly engaged with their goal and indicated as such through verbal self-talk (e.g., “I got this,” “Let’s do this”). However, without objective measurement of goal acceptance the level of engagement is unknown and a limitation. Finally, the design of the present study prevented randomization or counterbalancing. The number of field goals attempted in the timed condition established the number of field goals that would be attempted in the untimed condition, and the number of successful field goals from those conditions set the target for the goal-oriented condition. Thus, the order of condition was non-random, potentially introducing order effects. Allowing sufficient warmup and rest between conditions attempted to minimize these risks. Furthermore, the duration of shots and number of field goals attempted did not differ between the three conditions, indicating that there was no change in strategy between timed and goal-oriented conditions. However, these order effects are potential confounds to these findings and require additional research.

Despite these limitations, several important applied considerations remain. First, time constraints can and do increase anxiety and dissociate attention. This is particularly true in novice participants but is supported across skill levels. Second, goal setting is a simple psychological skill that benefits athletic performance in complex motor tasks under time constraints. Setting appropriate, realistic, and approach-oriented goals is an efficacious intervention for athletes performing under time demands. These positive effects of goal setting are particularly relevant for experienced athletes. Third, consistency in major joints angles was associated with improved performance. Athletes and coaches should consider training for movement consistency, especially for larger muscle groups. Fourth, for higher skill levels, performing under time pressure can increase athletic performance. Time constraints, in the absence of catastrophic levels of anxiety, may facilitate more automated motor movements.

## Data Availability Statement

The raw data supporting the conclusions of this article will be made available by the authors, without undue reservation.

## Ethics Statement

The studies involving human participants were reviewed and approved by the University of North Florida IRB. The patients/participants provided their written informed consent to participate in this study.

## Author Contributions

The author confirms being the sole contributor of this work and has approved it for publication.

## Conflict of Interest

The author declares that the research was conducted in the absence of any commercial or financial relationships that could be construed as a potential conflict of interest.

## Publisher’s Note

All claims expressed in this article are solely those of the authors and do not necessarily represent those of their affiliated organizations, or those of the publisher, the editors and the reviewers. Any product that may be evaluated in this article, or claim that may be made by its manufacturer, is not guaranteed or endorsed by the publisher.
